# Genetic characterization of antimicrobial resistance of *Shigella flexneri* 1c isolates from patients in Egypt and Pakistan

**DOI:** 10.1186/1476-0711-12-9

**Published:** 2013-05-02

**Authors:** Salwa F Ahmed, John Klena, Tupur Husain, Jesse Monestersky, Amel Naguib, Momtaz O Wasfy

**Affiliations:** 1 Research Science Directorate, United States Naval Medical Research Unit No. 3, Cairo, Egypt; 2 Department of Epidemiology, Ministry of Health and Population, Cairo, Egypt

**Keywords:** *S. flexneri* 1c, Genetic diversity, Antibiotic resistance, Oxa1, PFGE, Egypt, Diarrhea, Pakistan

## Abstract

**Background:**

*Shigella flexneri* serotype 1c emerged as a critical isolate from children in Egypt and Pakistan. The pattern of antimicrobial susceptibility (AMS) and resistance genes of this serotype have yet to be characterized.

**Findings:**

Sixty nine *S. flexneri* 1c isolates isolates were identified from both Egypt (n-46) and Pakistan (n = 23) and tested for AMS by disk diffusion method and minimal inhibitory concentrations were also determined. Isolates were genotyped by pulsed field gel electrophoresis (PFGE) and five relevant resistance genes (bla_TEM_, bla_SHV_, bla_OXA_, *sul*I and *sul*II) were detected by polymerase chain reaction (PCR) and confirmed by DNA sequencing. High resistance was observed in all isolates for ampicillin (AM >96%); trimethoprim-sulphamethoxazole and tetracycline (>88%). Most AM-resistant isolates from Egypt (70%) harbored bla_TEM_ resistance, while 52% of isolates from Pakistan expressed *bla*_OXA_. All isolates were closely related by PFGE, irrespective of source or time of collection. The *sul*II gene was present in 100% of isolates from pediatric cases in Egypt, 65% of Pakistan isolates, and 53% of isolates from older Egyptian patients.

**Conclusions:**

While different *Shigella* serotypes gathered in specific genotypic groups, 1c serotype isolates formed multiple clusters. Although AMS was considerably high to most commonly used drugs, genetic determinants were variable between countries over time. The data stress the need for a more careful selection of antibiotics in the treatment of shigellosis.

## Findings

While *Shigella sonnei* is the main cause in industrialized countries, *S. flexneri* is predominant in developing countries (particularly serotype 2a) [1-4]. In a recent study, *S. flexneri* serotype 1c emerged as an important isolate (17%) in three pediatric populations in Egypt [5]. The isolates were highly resistant to AM, tetracycline and sulfatrimethoxazole, with some showing simultaneous resistance to two or more antibiotics (multi-drug resistance phenotypes, MDR). Outside Egypt*,* this serotype has been described in Southeast Asia and the Indian subcontinent [5-8]. Also, many untypeable *S. flexneri* isolates from Pakistan belonged to serotype 1c [9]. The purpose of the present study was to characterize this serotype in a collection of isolates from Egypt and Pakistan for phenotypic and molecular markers of resistance to β-lactams and sulfonamides.

A total of 46 *S. flexneri* serotype 1c isolates from Egypt were used; 10 from pediatric patients (EP, <5 years) examined between 1990 and 1994, 17 between 2000 and 2004 and 19 between 2001 and 2003. The last group came from non-pediatric patients (NEP; 9–19 years). Nineteen isolates were also obtained between 2002 and 2004 from Pakistani pediatric (PP) clinics located in the urban slums of Karachi [9,10]. All isolates were cultured from rectal swabs and/or stool samples and identified at NAMRU-3 using standard microbiological and biochemical procedures [11]. Informed consents were obtained from all involved adults and guardians of minors. The study was approved by the Institutional Review Board (IRB Protocol No 96) of NAMRU-3, in compliance with Helsinki Declaration and US Federal regulations governing the protection of human subjects. Species serotypes were determined using commercially available serotyping kits [5], whereas the identity of serotype 1c was confirmed using monoclonal antibodies specific for *S. flexneri* 1 c (MASF) (obtained through the courtesy of Dr. Carlin, 1989). Antimicrobial susceptibility testing (AMS) was performed against 9 types of antibiotics ampicillin (AM, 10 ug), tetracycline (TE, 30 ug), trimethoprim-sulphamethoxazole (SXT, 25 ug), chloramphenicol (C, 30 ug), ciprofloxacin (CIP, 5 ug), ceftriaxone (CRO, 30 ug), cefepime (FEB, 30 ug), ceftazidime (CAZ, 30 ug) and cefotaxime (CTX, 30 ug) by the disk diffusion and E-strips methods as previously described [12]. MDR isolates were defined as those showing simultaneous resistance to at least three antibiotics [5]. Detection of β-lactam genes was performed by amplifying specific regions of the bla_TEM_, bla_SHV_, and bla_OXA_ genes and the mechanism of resistance to sulfa compounds was determined by amplifying the genes *sul*I and *sul*II [13]. Testing for class 1 and 2 integrons was also conducted [13,14]. Primers were commercially purchased from Sigma-Genosys (St Louis, MO.USA). PCR amplicons were sequenced and confirmed as shown previously [13]. Genomic DNA was prepared to conduct Pulsed Field Gel Electrophoresis (PFGE) [15] using XbaI mrp and the obtained phylogenetic tree was analyzed at a similarity level of 95% [5].

## Availability of supporting data

High levels of drug resistance were found among and within patient groups in both Egypt and Pakistan (Table 1). The majority of isolates (≥88%) were resistant to AM and TE, with expected minimal inhibitory concentrations > 16 ug/ml [3]. Also, 83% of PP isolates were resistant to C (≥ 32 ug/ml), while recent EP and NEP isolates were susceptible. Resistance to SXT was only 20% in EP 1990–1994 isolates and increased considerably to 88% in EP 2000–2004 and NEP isolates (89%) (p > 0.05). In PP isolates, however, resistance to this drug was 96%. MDR phenotype AM/C/SXT reached 83% in PP isolates and ranged from 11 to 20% in EP 1990–1994 and NEP isolates. Other MDR phenotypes (AM/TE/SXT) were found in 79-82% of EP 2000–2004, NEP and PP isolates. While all isolates from both countries were susceptible to CRO, FEB, CAZ and CTX, some (average = 31 ± 35%) showed resistance to cephalothin.

**Table 1:** **Antibiotic resistance profile of *S. flexneri *1c isolates from Egypt and Pakistan using both disk diffusion and E-test methods**

**Antibiotics**	**AM**^**1**^			**C**			**TE**			**SXT**			**CET**			**CIP**			**CRO**			**FEB**			**CAZ**			**CTX**		
	**S**^**2**^	**I**	**R**	**S**	**I**	**R**	**S**	**I**	**R**	**S**	**I**	**R**	**S**	**I**	**R**	**S**	**I**	**R**	**S**	**I**	**R**	**S**	**I**	**R**	**S**	**I**	**R**	**S**	**I**	**R**
CLSI MIC^3^ breakpoints μg/mL	≤8	16	≥32	≤ 8	16	≥ 32	≤4	8	≥16	≤2/38		≥4/76	≤8	16	≥32	≤1	2	≥4	≤1	2	≥4	≤8	16	≥32	≤4	8	≥16	≤1	2	≥4
EP^4^ isolates (n = 10) (1990–1994)	0	0	10 (100)^7^	0	0	10 (100)	0	0	10 (100)	6 (60)	2 (20)	2 (20)	0	2 (20)	8 (80)	10 (100)	0	0	ND^8^			ND			ND			ND		
EP isolates (n = 17) (2000–2004)	1 (6)	0	16 (94)	16 (94)	0	1 (6)	2 (12)	0	15 (88)	2 (12)	0	15 (88)	1 (6)	14 (82)	2 (12)	17 (100)	0	0	17 (100)	0	0	17 (100)	0	0	17 (100)	0	0	17 (100)	0	0
NEP^5^ isolates (n = 19) (2001–2003)	0	0	19 (100)	16 (84)	0	3 (16)	0	0	19 (100)	2 (11)	0	17 (89)	1 (5)	12 (63)	6 (32)	19 (100)	0	0	19 (100)	0	0	19 (100)	0	0	19 (100)	0	0	19 (100)	0	0
PP^6^ isolates (n = 23) (2002–2003)	1 (4)		22 (96)	4 (17)		19 (83)	2 (9)		21 (91)	1 (4)		22 (96)	6 (26)	17 (74)	0	10 (100)	0	0	23 (100)	0	0	23 (100)	0	0	23 (100)	0	0	23 (100)	0	0

^1^*AM* Ampicillin, *C* Chloramphenicol, *TE* Tetracycline, *SXT* Trimethoprim sulfamethoxazole, *CET* Cephalothin, *CIP* Ciprofloxacin, *CRO* Ceftriaxone, *FEB* Cefepime, *CAZ* Ceftazidime, *CTX* Cefotaxime.

^2^*S*, *I* and *R* Susceptible, Intermediate and Resistant.

^3^*MIC* Minimum inhibitory concentrations.

^4^*EP* Egyptian pediatric isolates (<5 years).

^5^*NEP* Non-pediatric Egyptian isolates (9–19 years).

^6^*PP* Pakistan pediatric isolates (<5 years).

^7^ () % of isolates.

^8^*ND* Not done.

**Figure 1:**
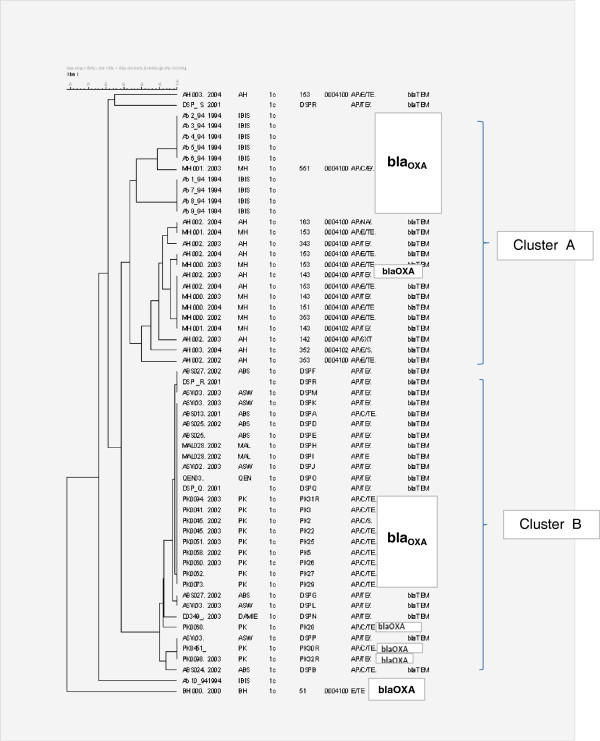
**The source of the isolate is provided by a two letter code followed by a unique identifying number; an underscore separates the year of isolation from the identifying number.** Source abbreviations: AH, Abu Homos, BH, Benha; MH, Mokattum Hills. PK, Pakistan; QEN, ASW, MAL, ABS, P, D, Q,R, S, different regions from upper Egypt and Cairo. Antibiotic resistance profile abbreviations are as follows: AM, ampicillin resistance; C, choramphenicol resistance; E, erythromycin resistance; TE, Tetracycline resistance; SXT, trimethoprim-sulfamthoxazole resistance. Clonal distance is shown after whole genomic digestion using the restriction enzyme xbaI. All samples were normalized against the pattern of Salmonella enterica ss. Enterica serotype Braenderup strain H9812. Band pattern analysis was performed using Bionumerics vs 4.5 and dendrogram was calculated using Dice similarity coefficients, based on the unweighted pair group method with arithmetic averages (UPGMA).

Of Egypt’s EP and NEP AM-resistant isolates, 70% harbored Bla_TEM-1_ resistance gene and the remaining carried Bla_OXA-1_ gene. None of the PP isolates harbored the Bla_TEM −1_ gene while only 52% of AM-resistant isolates expressed bla_OXA-1_ gene. The gene *sul*II was detected in 10% of EP 1990–1994 isolates, 53% of NEP and 65% of PP isolates. However, it was found in all EP 2000–2004 isolates. The *sul*I gene was associated with bla_OXA_-1 gene in 48% of the isolates. Out of the bla_TEM_ positive isolates, 72% (23/32) were positive for class 2 integron and 6% were positive for class 1 integron. The proportions of integrons among bla_OXA_-positive cases were relatively similar. EP and PP isolates that failed to amplify the Bla_OXA_ and Bla_TEM_ genes were negative for Bla_CTXM_, bla_CMY_, and bla_SHV_[13]. XbaI mrp-PFGE grouped the majority of isolates in two major clusters; cluster A included 90% of EP isolates positive for bla_OXA_ and cluster B covered most NEP isolates that harbored bla_TEM_ gene and PP isolates with bla_OXA_ gene (Figure 1).

The recent emergence of *S. flexneri* 1c as a dominant serotype of this species was reported in Southeast Asia and the Indian subcontinent [6-8]. In Egypt, this serotype was identified in three different pediatric populations and their antimicrobial susceptibility patterns demonstrated a trend towards multi-drug resistance, with a dominant resistance phenotype against AM, TE, and SXT. In the present study, more *S.flexneri* serotype 1c isolates from Egypt were characterized by phenotypic and molecular methods in comparison with a group of isolates obtained from a pediatric study in Pakistan.

While the obtained data may provide a useful baseline for AMS patterns and underlying genes, further studies to evaluate more recent isolates are underway. The high levels of antimicrobial resistance to many drugs in Egypt and Pakistan is in consent with previous reports [16-18] and reflects common medical treatment practices in both countries. Accordingly, CRO has been recommended as a first-line parenteral therapy for children with shigellosis [19] and CIP was used as an alternative when CRO is contraindicated [20,21]. The increased SXT resistance in EP and PP isolates suggests a wider use of SXT treatment during the late 90s in Egypt and Pakistan [18] and exemplifies a dynamic selective drug pressure influence on AMS patterns [22]. However, a trend towards intermediate resistance to cephalothin has been noticed among all *Shigella* isolates in this study, which is probably an early alert against the development of resistance to third generation cephalosporins. This observation may suggest leaving this line of drug for empirical use in serious cases of shigellosis only [10]. While EP isolates collected between 1990 and 1994 showed 20% of MDR phenotype AM/C/SXT, those collected between 2000–2004 demonstrated 82% of MDR phenotype AM/TE/SXT (Table 1), suggesting that some of the tested drugs may be of limited use as first line therapy [10]. Although little has been known about the prevalence of extended spectrum beta lactamases in *S. flexneri* serotype 1c isolates, molecular analysis of resistance genes revealed substantial differences among the studied populations with a predominance of bla_TEM_ in EP and NEP strains, possibly due to a single gene copy on the large conjugatively transferable plasmid known to endow resistance to AM and SXT [23]. However, the remaining EP isolates carried the bla_OXA_ gene, similar to approximately half of the Pakistan isolates (12/23). Previous studies have demonstrated the presence of bla_OXA_ gene in AM resistant *S.flexneri* in different geographic areas including Denmark [24] and Tanzania [25].

In developing countries, SXT is still used as a first-line antibiotic. Yet, 79% (55/69) of the current 1c isolates were resistant to this drug. The *dhfr*Ia gene which is transferred through a class 2 integron was implicated in SXT resistance [26]. The detection of class 2 integron in 63% of all isolates probably portrays its role as a mobile element in the dissemination of antibiotic resistance to SXT and C [23]. In the present study, nucleotide sequence analyses revealed that all the bla_OXA_ genes produced OXA-1 type B-lactamase. Interestingly, 88% of the bla_OXA_-1 isolates from NPE and PP demonstrated resistance to C, although some studies have reported that C resistance is chromosomally mediated through the *catA1* gene (8). An association between bla_OXA-1_ gene and AM resistance among *Shigella* isolates has been documented [24,25] owing to the presence of a class 1 integron that may be placed on the chromosomes to counteract the tendency of losing the virulence/resistance plasmid [8]. However, the susceptibility of isolates to CRO, FEB, CAZ and CTX provides promising treatment options.

Regardless of time, genetic elements mediating resistance and location, XbaI mrp-PFGE profiles placed the majority of *S.flexneri* 1c isolates in more than one cluster, unlike other *S.flexneri* serotypes 1b, 2a, 3, 4, and 6 that may show unique clusters for each serotype (5). PFGE grouped 90% of infant EP isolates collected between 1990 and1994 in one cluster, indicating minor genetic differences. Meanwhile, NEP and PP isolates formed another cluster, probably in relation to age of the patients.

The obtained data highlight the emergence of *S. flexneri* 1c as an additional serotype in endemic areas. It carries and possibly disseminates drug resistance through variable genetic mechanisms over time and location and should not be overlooked. The spread of multi-resistant strains of this serotype among pediatric and adult populations in endemic areas supports the need for a wiser selection and use of antibiotics. Plans for its proper identification, treatment and control are certainly more urgent than ever.

### Consent

Written informed consent was obtained voluntarily from all adult patients and guardians of children before participation or enrollment in the study in keen for publication of this report.

## Abbreviations

AMS: Antimicrobial susceptibility; MDR: Multi-drug resistance phenotypes; EP: Egyptian pediatric patients; NEP: Egyptian non-pediatric patients; PP: Pakistani pediatric; AM: Ampicillin; TE: Tetracycline; SXT: Trimethoprim-sulphamethoxazole; C: Chloramphenicol; CIP: Ciprofloxacin; CRO: Ceftriaxone; FEB: Cefepime; CAZ: Ceftazidime; CTX: Cefotaxime; PFGE: Pulsed Field Gel Electrophoresis

## Competing interests

The authors declare that they have no competing interests.

## Authors’ contributions

SFA carried out the molecular genetic studies, wrote the manuscript. JK participated in the design of the study, help in drafting the manuscript, TH coordinated and helped to draft the manuscript. JM critical revision of manuscript and data content. AN provided final approval for publication. MOW performed data analysis, results interpretation and helped in writing the manuscript. All authors read and approved the final manuscript.

## Authors’ information

Co-authors: John Klena, Tupur Husain, Jesse Monestersky, Amel Naguib, Momtaz O Wasfy.

## References

[B1] Ahmed F Clemens JD Rao MR Ansaruzzaman M Haque E Epidemiology of shigellosis among children exposed to cases of Shigella dysentery: a multivariate assessment AmJTrop Med Hyg 1997 56 258 264 10.4269/ajtmh.1997.56.258 9129527

[B2] Ferreccio C Prado V Ojeda A Epidemiologic patterns of acute diarrhea and endemic Shigella infections in children in a poor periurban setting in Santiago, Chile Am J Epidemiol 1991 1134 614 627 1951266 10.1093/oxfordjournals.aje.a116134

[B3] Joe LK Rukmono B Oemijati S Diarrhoea among infants in a crowded area of Djakarta, Indonesia. A longitudinal study from birth to two years Bull World Health Organ 1996 34 197 210 5296127 PMC2475927

[B4] Kotloff KL Winickoff JP Ivanoff B Global burden of Shigella infections: implications for vaccine development and implementation of control strategies Bull World Health Organ 1999 77 651 666 10516787 PMC2557719

[B5] Ahmed SF Riddle MS Wierzba TF Abdel-messih I Monteville MR Sanders JW Klena JD Epidemiology and genetic characterization of Shigella flexneri strains isolated from three paediatric populations in Egypt (2000–2004) Epidemiol Infect 2006 134 1237 1248 10.1017/S095026880600642X 16690004 PMC2870525

[B6] Kosek M Yori PP Olortegui MP Shigellosis update: advancing antibiotic resistance, investment empowered vaccine development, and green bananas Curr Opinion Infect Dis 2010 23 475 480 10.1097/QCO.0b013e32833da204 PMC6892429 20689423

[B7] Stagg RM Cam PD Verma NK Identification of newly recognized serotype 1c as the most prevalent *Shigella flexneri* serotype in northern rural Vietnam Epidemiol Infect 2008 136 1134 1140 17922932 10.1017/S0950268807009600 PMC2870902

[B8] Talukder KA Islam Z Islam MA Dutta DK Safa A Ansaruzzaman M Faruque AS Shahed SN Nair GB Sack DA Phenotypic and genotypic characterization of provisional serotype *Shigella flexneri* 1c and clonal relationships with 1a and 1b strains isolated from Bangladesh J Clin Microbiol 2003 41 110 117 10.1128/JCM.41.1.110-117.2003 12517835 PMC149623

[B9] Zafar A Hasan R Nizami SQ Von Seidlein L Soofi S Ahsan T Chandio S Habib A Bhutto N Siddiqui FJ Rizvi A Clemens JD Bhutta ZA Frequency of isolation of various subtypes and antimicrobial resistance of Shigella from urban slums of Karachi, Pakistan Int J Infect Dis 2009 13 668 672 10.1016/j.ijid.2008.10.005 19135399

[B10] Zafar A Sabir N Bhutta ZA Frequency of isolation of *Shigella* serogroups/serotypes and their antimicrobial susceptibility in children from slum areas in Karachi J Pakist Med Assoc 2005 55 184 188 15960281

[B11] Wierzba TF Abdel-Messih IA Abu-Elyazeed R Putnam SD Kamal KA Rozmajzl P Ahmed SF Fatah A Zabedy K Shaheen HI Sanders J Frenck R Clinic-based surveillance for bacterial- and rotavirus-associated diarrhea in Egyptian children AmJTrop Med Hyg 2006 74 148 153 16407360

[B12] Clinical and Laboratory Standards Institute (CLSI) Performance standards for antimicrobial susceptibility testing; twentieth informational supplement Performance standards for antimicrobial susceptibility testing; twentieth informational supplement (document M 100-S20) 2010 Wayne, PA; USA: CLSI document M 100-S20

[B13] Fam N Leflon-Guibout V Fouad S Aboul-Fadl L Marcon E Desouky D El-Defrawy I Abou-Aitta A Klena J Nicolas-Chanoine MH **CTX-M-15-producing *****Escherichia coli *****clinical isolates in Cairo (Egypt), including isolates of clonal complex ST10 and clones ST131, ST73, and ST405 in both community and hospital settings.** Microb Drug Resist 2011 17 67 73 10.1089/mdr.2010.0063 21128836

[B14] El-Gendy AM Mansour A Weiner MA Pimentel G Armstrong AW Young SYN Elsayed N Klena JD Genetic diversity and antibiotic resistance in *Shigella dysenteriae* and *Shigella boydii* strains isolated from children aged < 5 years in Egypt Epidemiol Infect 2012 140 299 310 10.1017/S0950268811000525 21470441

[B15] Hunter SB Vauterin P Mary Ann Lambert-Fair MA Van Duyne MS Kubota K Graves L Wrigley D Barrett T Ribot E Establishment of a universal size standard strain for use with the PulseNet standardized pulsed-field gel electrophoresis protocols: converting the national databases to the new size standard J Clin Microbiol 2005 43 1045 1050 10.1128/JCM.43.3.1045-1050.2005 15750058 PMC1081233

[B16] Putnam SD Frenck RW Riddle MS El-Gendy A Taha NN Pittner BT Abu-Elyazeed R Wierzba TF Rao MR Savarino SJ Clemens JD Antimicrobial susceptibility trends in *Campylobacter jejuni* and *Campylobacter coli* isolated from a rural Egyptian pediatric population with diarrhea Diag Microbiol Infect Dis 2003 47 601 608 10.1016/S0732-8893(03)00154-8 14711482

[B17] Putnam SD Riddle MS Wierzba TF Pittner BT Elyazeed RA El-Gendy A Rao MR Clemens JD Frenck RW Antimicrobial susceptibility trends among *Escherichia coli* and *Shigella* spp isolated from rural Egyptian paediatric populations with diarrhoea between 1995 and 2000 Clin Microbiol Infect 2004 10 804 810 10.1111/j.1469-0691.2004.00927.x 15355411

[B18] Wasfy MO Oyofo BA David JC Ismail TF el-Gendy AM Mohran ZS Sultan Y Peruski LF Jr Isolation and antibiotic susceptibility of *Salmonella*, *Shigella*, and *Campylobacter* from acute enteric infections in Egypt J Heath Popul Nutr 2000 8 33 38 11014768

[B19] Eidlitz-Marcus T Cohen YH Nussinovitch M Elian I Varsano I Comparative efficacy of two- and five-day courses of ceftriaxone for treatment of severe shigellosis in children J Pediatr 1993 123 822 824 10.1016/S0022-3476(05)80868-6 8229499

[B20] Salam MA Dhar U Khan WA Bennish ML Randomized comparison of ciprofloxacin suspension and pivmecillinam for childhood shigellosis Lancet 1998 352 522 527 10.1016/S0140-6736(97)11457-X 9716056

[B21] Guidelines for the control of shigellosis, including epidemics due to Shigella dysenteriae 1 http://whqlibdoc.who.int/publications/2005/9241592330.pdf

[B22] McCormick AW Whitney CG Farley MM Lynfield R Harrison LH Bennett NM Schaffner W Reingold A Hadler J Cieslak P Samore MH Lipsitch M Geographic diversity and temporal trends of antimicrobial resistance in Streptococcus pneumoniae in the United States Nature Medicine 2003 9 424 430 10.1038/nm839 12627227

[B23] Siu LK Lo JY Yuen KY Chau PY Ng MH Ho PL β-lactamases in *Shigella flexneri* isolates from Hong Kong and Shanghai and a novel OXA-1-like β-lactamase, OXA-30 Antimicrob Agents Chemother 2000 44 2034 2038 10.1128/AAC.44.8.2034-2038.2000 10898672 PMC90010

[B24] Schumacher H Nir M Mansa B Grassy A β-Lactamases in *Shigella* APMIS 1992 100 954 956 10.1111/j.1699-0463.1992.tb04024.x 1445702

[B25] Navia MM Capitano L Ruiz J Vargas M Urassa H Schellemberg D Gascon J Vila J **Typing and characterization of mechanisms of resistance of Shigella *****spp *****isolated from feces of children under 5 years of age from Ifakara, Tanzania.** J Clin Microbiol 1999 37 3113 3117 10488163 10.1128/jcm.37.10.3113-3117.1999 PMC85506

[B26] Toro CS Farfán M Contreras I Flores O Navarro N Mora GC Prado V Genetic analysis of antibiotic-resistance determinants in multidrug-resistant Shigella strains isolated from Chilean children Epidemiol Infect 2005 133 81 86 10.1017/S0950268804003048 15724714 PMC2870225

